# Competencies for on‐call physicians for home medical care: A qualitative study of home care providers' experiences in Japan’s super‐aged society

**DOI:** 10.1002/jgf2.542

**Published:** 2022-03-30

**Authors:** Hiroyuki Nagano, Yukio Tsugihashi, Mai Tatsuno

**Affiliations:** ^1^ Department of General Internal Medicine Tenri Hospital Tenri City, Nara Japan; ^2^ Medical Home Care Center Tenri Hospital Shirakawa Branch Tenri city, Nara Japan; ^3^ Department of Field Medicine, Graduate School of Medicine Kyoto University Kyoto city, Kyoto Japan

**Keywords:** competency, home medical care, on‐call physician, on‐call task, semi‐structured interview

## Abstract

**Background:**

In Japan, many older people hope to receive end‐of‐life care at home. In such situations, team‐based home medical care with the support of on‐call physicians is needed. However, to date, necessary competencies for the on‐call physicians have yet to be clarified. We aim to reveal the competencies for on‐call physicians in home medical care settings.

**Method:**

This was a qualitative study of semi‐structured interviews concerning competencies for on‐call physicians in home medical care. We evaluated digitally recorded interviews with eight home care professionals in seven home care facilities (three clinics, one hospital, and three nursing agencies) in A City, Japan. The transcribed data were analyzed by three researchers using thematic analysis.

**Results:**

The competencies for on‐call physicians were divided into the following six categories: clinical skills for frequent complaints, collecting patients' information in advance, understanding purposes of home care, understanding general roles of home care health professionals, thoughtfulness toward patients' families, and an attitude of humility.

**Conclusion:**

These competencies were classified into disease‐specific and interpersonal (collaborative) skills in home medical care. The competencies revealed by the study could contribute to the development of effective learning and preparation for on‐call physicians who support home medical care.

## Graphical abstract

This was a qualitative study of semi‐structured interviews concerning competencies for on‐call physicians in home medical care. These competencies were classified into disease‐specific and interpersonal (collaborative) skills in home medical care. The competencies revealed by the study could contribute to the development of effective learning and preparation for on‐call physicians who support home medical care.

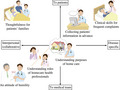

## 1 INTRODUCTION

Japan is experiencing an unprecedented level of population aging. In 2012, Japan was the only country where the proportion of people aged 60 years or older exceeded 30%. By 2030, nearly one in every three people will be 65 years or older, and one in five people will be 75 years or older.^1^ Moreover, in many countries such as Europe, North America, and Asia, the proportion of people aged 60 years or older will exceed 30% by 2050.^2^

Older people need home care services because of functional decline and multimorbidity.^3^ In addition, a nationwide survey revealed that more than 60% of Japanese hoped to receive end‐of‐life care at home.^4^ The need for home care physicians is estimated to increase to 1.7 times in Japan in the late 2030s.^5^ To facilitate home medical care, the Japanese government has promoted the home medical care for disabled elderly people with cognitive function decline.^6^ Therefore, a robust home medical care system with 24‐h availability is needed to prepare for the increasing needs of older patients requiring home medical care, including those in their end‐of‐life stages. Based on another survey in Japan, however, over 70% of physicians provided home medical care (home care physicians) in solo practice and were struggling to provide 24‐h care.^7^

To maintain the provision of 24‐h home medical care, team‐based care comprising multidisciplinary professionals is necessary. As for the team of home care physicians, cooperation with part‐time physicians could also be of great support in reducing the burden of on‐call work. One study in Japan showed that part‐time physicians accounted for 26.2% of all home care physicians and over 60% of part‐time physicians were under 40 years old.^8^ Moreover, residents of the internal medicine and the general practice could have more opportunities to be involved in home medical care and on‐call tasks in their residency programs. This encourages clinical training in community health care, including home medical care.^9^, ^10^ When part‐time physicians and residents participate in on‐call duty, they want to know the essential competencies for home medical care that are expected by professional home care teams. *Competency* in medical education is defined as a learnable, durable, and measurable ability to execute a specific, integrative task,^11^ and competency‐based training enable part‐time physicians to know what they need to learn, specific to home medical care.^12^ In 2003, the Committee on the Health Professions Education Summit proposed core competencies for healthcare professionals, which consist of providing patient‐centered care, working in interdisciplinary teams, employing evidence‐based practice, applying quality improvement, and utilizing informatics.^13^ Focusing on home medical care, the Japanese Association for Home Care Medicine also proposed the following competencies for home care physicians: professionalism, continual support, comprehensive support, family support and adjustment of care environment, community‐based activity, and contribution to the improvement of quality of home medical care.^14^ However, the competencies for on‐call physicians in home medical care have not yet been studied. Contrary to the usual home medical care, on‐call physicians might treat patients at home without trusting relationships. In addition, on‐call physicians are unlikely to have the opportunity to consult with other professionals during their shifts. Therefore, the purpose of this study was to identify and confirm the required competencies for on‐call physicians in home medical care.

## 2 METHOD

### 2.1 Design

This study performed a qualitative thematic analysis of semi‐structured interviews.^15^

### 2.2 Participants

This study enrolled all home care institutions that participated in information and communication technology (ICT) networks operated by the Medical Association and usually provided coordinated team‐based home medical care in City A, Japan. The ICT networks in City A were developed for multidisciplinary information sharing in a home medical care setting. There are patients' information, medical information, and photos necessary for multidisciplinary collaboration in the ICT network. All the institutions providing home medical care (three clinics and one hospital) and nursing care (three nursing facilities and one hospital) approved our interview request. All representative home healthcare physicians (*n* = 4) and nurses (*n* = 4) from each facility completed a semi‐structured interview in their offices. These facilities do not hire part‐time physicians performing on‐call tasks. Target home care professionals were not included in the team of interviews and analysis.

### 2.3 Setting

A city had a population of 66,800 in 2016, one‐quarter of which was aged 65 years or older. In 2016, 160 patients were required home medical care by home care physicians in the three clinics and one hospital. Two hundred and eighty‐five patients were supported through home nursing care in City A.^16^

### 2.4 Definition

In this research, we defined on‐call physicians who are responsible for on‐call tasks such as responding to patients' complaints and consultations from home care health professionals after hours. On‐call physicians do not visit patients regularly and do not make business decisions, such as coordinating the visiting schedules or patient numbers.

### 2.5 Interviews

This study performed semi‐structured interviews to identify competencies for on‐call physicians for home medical care, which were required by the participants. All interviews were carried out in the participants' workplaces by a researcher with a master’s degree in interviewing or analyzing qualitative data (the second author) and hospital physicians who have experienced monthly training in home medical care during their senior residence internal medicine programs (either of the first and third authors). All interviewers reviewed the semi‐structured questions before conducting the interviews. The interviews were conducted according to an interview guide (Table 1), and additional questions were asked if the interviewers needed further information especially from the perspective of hospital physicians. Consent for participation and for being digitally recorded was obtained before the interviews, and all interviews were recorded and transcribed into verbatim records. Each interview took appropriately 30–60 min.

**Table 1:** The interview guide

The aim of this interview is to understand core competencies for on‐call physician for home medical care.
We defined on‐call physicians as physicians who are responsible for on‐call tasks in homecare settings.
<Questions in the interview>
1. What kind of symptoms and situations does an on‐call physician respond to?
2. What kind of countermeasures do you expect from an on‐call physician?
3. What are the abilities and knowledge are necessary for on‐call physicians?
4. What kind of information should be shared in order to take appropriate action?
5. In what form do you think it is desirable to share this information?
Additional questions were asked if the interviewers needed further information especially from the perspective of hospital physician.

### 2.6 Analysis

The interviews were analyzed using thematic analysis by the three researchers (the first, second, and third authors).^17^ The researchers read through the entire verbatim records of the interviews several times and took notes on information relevant to our research question. Moreover, we divided the text records into a dataset with meaningful contents with code numbers from 1 to 165 (File S1). The codes were categorized into subthemes based on similarities of meaning. Through a discussion among the authors, we refined the themes and selected the most representative competencies in relation to our research question. This research was approved by the ethical committee of B Hospital (Approval number: 776, 779).

## 3 RESULTS

In this study, we identified a total of six competencies for on‐call physicians in home medical care from the 165 codes as shown in Table 2.

**Table 2:** Six competencies for on‐call physicians in home medical care

Competency	Representative comments (code number*)
Clinical skills for frequent complaints	“Fever constitutes the majority of problems in home visit. Pneumonia and UTI account for about 90% of fever cases”. (137)
	“I’d like to ask on–call physicians to certify death at home when home care physician is on vacation or I can’t get in contact with him.” (153)
	“We should deal with catheter troubles such as nasogastric tube, urinary catheter and percutaneous gastrostomy tube.” (146)
Collecting patients' information in advance	“It is important to share information about patients such as their medical history, their personality and the relationship between patients and their families.” (109)
	“The home care physician and on‐call physician should share information about the patients' prognosis, therapeutic plan and their expectation.” (106)
	“It is safe that on‐call physicians go for home‐visits with home care physician beforehand. They can not only see the style of home care physician but also make relationship with home care physician and patients.” (102)
Understanding purposes of home care	“Home care is different from hospital medicine. Medicine in home care must not disturb the lives of patients and families.” (20)
	“In home medical care, health care providers are visitors in the patients' house.” (19)
	“How to communicate with patients well is most important at hospital. It’s same with home care medicine.” (16)
Understanding roles of home care health professionals	“Home care nurses understand the policies of family doctors and have a lot of information about the patients at home.” (123)
	“Home care nurses get more information about what patients and families feel than doctors.” (126)
	“We tell the patients that first they try to contact with home care nurse when they have problems.” (125)
Thoughtfulness for patients' families	“The communication skills with patients and their families are important in home care because on‐call physicians provide medical care at patients' home and cannot perform examinations like in hospitals.” (58)
	“On‐call physicians should tell patients and their families that they communicate with home care physician fully to reassure them.” (45)
	“In end of life, patients and their families feel uncertain. Doctors should understand their feeling and communicate with them.” (47)
An attitude of humility	“I want on‐call physicians to understand and respect the policies of family doctors and treat patients based on the policies.” (7)
	“On‐call physicians should understand what kinds of treatment are needed in urgent visits and focus on urgent care.” (8)
	“In home care, mindsets of patient’s families change dramatically. Understanding and coping with their mindset isn’t an on‐call physician’s task, but home care physician’s one, I think.” (12)

Code numbers were assigned to the dataset with meaningful sentences or contents of the text recorded in the interviews.

### 3.1 Clinical skills for common complaints

On‐call physicians are required to prepare for complaints common in home care settings. Although problems common in hospital settings, such as fever and pain control, were also frequent complaints among home care patients, home care physicians must treat their patients without diagnostic equipment such as X‐rays, CT scans, and timely laboratory findings. In addition, home care physicians must handle medical devices such as nasogastric tubes, percutaneous gastrostomy tubes, and urinary catheters because homebound patients require immediate assistance in cases of accidental removal or occlusion of the tubes. Furthermore, home care physicians are responsible for issuing death certificates and completing required paperwork in patients' homes without any assistance from office workers.

### 3.2 Collecting patients' information in advance

On‐call physicians can collect patients' information with home care physicians and nurses before making urgent home visits. For instance, on‐call physicians can gather patient information including medical and nonmedical information such as patients' personalities and family relationships efficiently through home care physicians and nurses, and by searching medical records. Further, some home care physicians advised on‐call physicians to join them, preceding their on‐call tasks, to visit unstable patients who have potential needs for urgent house calls with experienced home care physicians. These home visits could contribute to the on‐call physicians' understanding of the home care physicians' style of practice and to the forging and fostering of relationships with patients and families, which are helpful during urgent visits.

### 3.3 Understanding the purposes of home care

On‐call physicians need to understand the differences between home medical care and hospital medicine. Home medical care must not interfere with the daily lives of patients and their families. For example, on‐call physicians should avoid forcing family caregivers to monitor vital signs frequently; night‐time monitoring, for example, which is common in hospitals, can be burdensome for the family caregivers. Moreover, patients’ perspectives and expectations must be respected in home medical care, as much as in hospital medicine. For example, communication with patients plays a key role in making clinical decisions in practice, whether in patients’ homes or hospitals. However, the patient‐centered perspective receives greater emphasis in home medical care because the place of treatment is the patients’ home, where patients and their families have control over their lives.

### 3.4 Understanding general roles of home care health professionals

Home care health professionals, especially nurses, play a coordinating role among home care teams, patients, and their families. They learn information about patients' hopes for treatment and future life through sustained engagement and maintaining a good relationship with them. For instance, the nurses sometimes receive information that patients are not comfortable sharing with their doctors. In addition, the nurses learned the home care physicians' methods of practice, such as palliative care. Therefore, on‐call physicians could access essential information about appropriate medical services by cooperating with home care nurses. Furthermore, the nurses usually triage the urgency and severity of patients' complaints and contact on‐call physicians when their assessment indicates the need for a home care physician’s immediate attention. Therefore, physicians on call should pay attention to nurses’ assessments to gather accurate and timely information about patients.

### 3.5 Thoughtfulness toward patients' families

On‐call physicians who provide home medical care should respect the emotional burden faced by the patients' family caregivers. In providing home‐based care, on‐call physicians should examine the patients carefully and communicate with them closely, since access to medical resources such as laboratory findings, imaging tests, and consultation with specialists in various fields is limited. Further, home care physicians should also pay attention to the emotional needs of family caregivers involved in end‐of‐life care, because the families tend to be anxious.

### 3.6 An attitude of humility

On‐call physicians should respect home care physicians' guidelines, despite any differences in policy and execution. For example, if there is a sudden change in the treatment plan during an urgent home visit by an on‐call physician, the patient, their families, and the nursing staff that attends to them may be confused and feel anxious. Therefore, on‐call physicians should focus on managing urgent complaints and hand over tentative care responsibilities to the home care physicians who provide the usual medical care. Moreover, on‐call physicians should realize that they cannot comprehensively understand the mindsets of patients and their families during urgent home visits.

## 4 DISCUSSION

**Figure 1:**
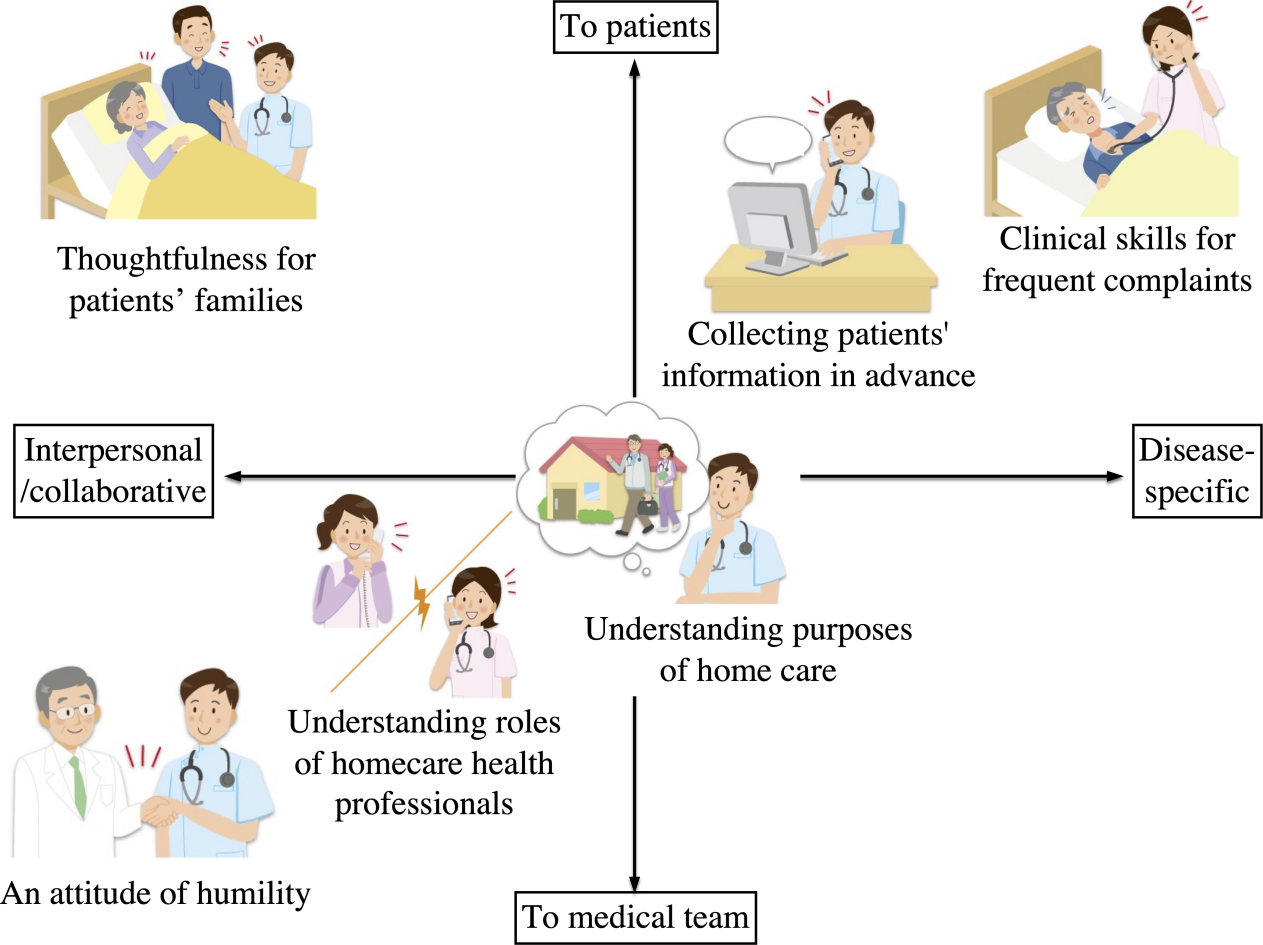
The categories of the six competencies for on‐call physicians in home medical care

This study was qualitative, and we analyzed themes derived from semi‐structured interviews to identify six competencies for on‐call physicians in home medical care: clinical skills for frequent complaints, collecting patients' information in advance, understanding the purposes of home care, understanding general roles of home care health professionals, thoughtfulness toward patients' families, and an attitude of humility. These competencies can be categorized into four areas using the following two axes: competencies regarding disease‐specific/interpersonal (collaborative) and those regarding patients/medical teams (Figure 1). As shown, the disease‐specific competencies regarding patients include clinical skills for frequent complaints and collecting patients' information in advance. Meanwhile, the interpersonal competencies regarding medical teams comprise understanding the roles of home care health professionals, and an attitude of humility. Thoughtfulness for patients' families is a disease‐specific/interpersonal competency concerning patients. Understanding the purposes of home care is centrally located among the four areas because it affects all components of the competencies.

As disease‐specific competencies, on‐call physicians should possess the clinical skills required for handling common problems related to home care. On‐call tasks do not always require difficult clinical skills; however, the on‐call physicians must independently perform the tasks under conditions where there is limited medical equipment. For example, physicians must provide palliative care during on‐call duty on their own, even if they usually prescribe opioids after consulting with specialists in‐hospital care. Moreover, they cannot quickly access equipment such as X‐ray, CT, and laboratory data. Additionally, they must deal with medical device‐related problems by themselves. For instance, on‐call physicians reinsert accidentally removed nasogastric or gastrostomy tubes, even if they are not accustomed to using such techniques in their daily practice. Certification of death is also an important on‐call task. In Japan, about 20% of all on‐call tasks involve certification of death.^16^ To cope with these urgent tasks during on‐call duty, physicians must become acquainted with the tasks in their daily work. If they do not have enough opportunities to experience procedures such as the insertion of gastrostomy tubes, simulation‐based learning is one of the options for gaining those skills.

Meanwhile, most of the competencies were also categorized into interpersonal (collaborative) competencies such as fundamental attitude, preparation, and communication with home care physicians. Similar types of competencies are found outside health care. For example, in the aviation industry, unsafe flight conditions were frequently related to failures in pilots' nontechnical (cognitive and social) skills, rather than a lack of knowledge and abilities.^18^ As commercial pilots fly as ad hoc rather than as part of a constituted crew, they must have good nontechnical skills for working as part of an effective team with unfamiliar members; therefore, there is a specialist‐training program called “crew resource management” to increase their use of nontechnical skills and improve safety. In medical fields such as anesthesia, surgery, and emergency medicine, there are similarities with the workload profiles of pilots (high intensity at task initiation and completion, monitoring, and rapid response to critical events with a rapidly formed team). An interpersonal skills framework was adopted and used for assessment, training, and education in such fields.^18^, ^19^ The SECTORS model is one of the frameworks supporting the design of effective nontechnical skills education in healthcare.^20^ It consists of three elements: core knowledge and skills, situated cognition (observation and experience), and analytical skills (risk assessment, situation awareness, and decision making). These elements are augmented by repeated observation, experience, assessment, and decision making in each situation. Our results can be applied to the core knowledge and skills aspect of this model, which can help teach nontechnical skills including interpersonal ones to home medical care providers. For instance, in the case of a terminal cancer patient, on‐call physicians could learn interpersonal skills according to the competencies such as “thoughtfulness toward patients' families” in advance. When providing on‐call services, physicians should realize the importance of listening to patients and their families. On‐call physicians could reflect on their practice throughout these experiences regarding the competencies after completing their tasks.

“An attitude of humility” is a unique competency for providing on‐call tasks in home medical care. What is “humility” in this case? Coulehan described *humility* as comprising three factors: (1) constant self‐awareness, (2) empathetic openness to others, and (3) keen appreciation of, and gratitude for, the privilege of caring for others.^21^ According to the interviews conducted during this research, the first and second factors are applicable here. Regarding self‐awareness, on‐call physicians should recognize what is needed from them in various situations. For example, one home care nurse said, “On‐call physicians should understand the type of treatment that is needed in urgent visits and focus on urgent care.” Concerning empathetic openness, on‐call physicians must respect the main physician’s vision and relationships with patients’ families and home care professionals. For instance, one home care nurse said, “I want on‐call physicians to understand and respect the policies of family doctors and treat patients based on the policies.” Considering these components of humility in home care settings, humility could be a fundamental competency for on‐call physicians who support home care physicians in developing strong relationships with their patients.

This is a novel qualitative study on the core competencies for on‐call physicians in home medical care. In Japan, part‐time physicians and residents have already been involved in on‐call tasks in home medical care. Clarifying these competencies could improve the quality of medical care by enabling efficient preparation and learning. These competencies could also reduce the technical and psychological barriers of nonspecialists in broadening their practice to home medical care. This study had some limitations. First, as this study was conducted in one community in Japan, it is unclear whether the findings can be generalized and applied to other communities with different healthcare systems or resources. However, this research is applicable in communities where there is a scarcity of home care physicians and where on‐call physicians are needed in home medical care. In some countries, a nurse practitioner is also involved in on‐call tasks in home medical care, and the six competencies identified in this research would also be useful for them. Second, this study had a small sample size, and the findings could not be theoretically saturated. However, we enrolled almost all healthcare institutions actively providing team‐based home care at the municipal level. Therefore, the findings from this study were an exhaustive set of competencies for on‐call physicians who entered team‐based home care in the community. To adapt the findings in other areas, the on‐call physician must consider the geographical characteristics, the number of medical resources, and the cultural background of the region based on the competencies. Third, these competencies are not suitable for solo practice by main home care physicians because this study focused on the on‐call duties provided by the other physicians. However, these competencies can be useful for those physicians who provide support to other home care physicians. Fourth, this study was performed before the COVID‐19 pandemic, and this result might not apply to the changed current home medical care situation including telehealth. Even in such special situations, interpersonal skills may be emphasized among the competencies we have identified^22^, ^23^, or other skills that have not been identified may be required. Fifth, most of these competencies have already been reported for healthcare professionals.^13^ However, this research first revealed competencies focusing on on‐call physicians in home medical care including a new concept such as “An attitude of humility.” Further research is needed to address these competencies by studying physicians who have experienced on‐call tasks in home medical care, in addition to surveys of home physicians and home nurses in other communities.

## 5 CONCLUSION

In conclusion, we suggest that on‐call physicians in home medical care possess six competencies classified as disease‐specific and interpersonal/collaborative. These competencies could contribute to effective preparation and learning for on‐call physicians who support home medical care.

## CONFLICT OF INTEREST

The authors have stated explicitly that there are no conflicts of interest in connection with this article.

## Supporting information


File S1
