# Supplementary material for: Competencies for on‐call physicians for home medical care: A qualitative study of home care providers' experiences in Japan’s super‐aged society
Source: J Gen Fam Med. 2022 Mar 30;23(4):261–7. doi: 10.1002/jgf2.542 (PMC9249937; doi:10.1002/jgf2.542)
Supplement: Supplementary file 1 — File S1 [file JGF2-23-261-s001.docx]

| **Coding number** | **Category of competency** | **Sub-theme** | **Interviewee** | **Meaningful contents** |
| --- | --- | --- | --- | --- |
| 1 | Understanding purposes of home care | Understanding the system of home medical care | nurse | Home care physicians and nurses provide medical care separately. |
| 2 | Understanding purposes of home care | Understanding the system of home medical care | physician | Physician and nurse visits should be carefully timed. |
| 3 | Understanding purposes of home care | Understanding the system of home medical care | nurse | If communication is effective, home care by a physician and home nursing can be provided on different days. |
| 4 | Understanding purposes of home care | Understanding the system of home medical care | nurse | Long-term care insurance cannot be used if the word “terminal” is indicated in the name of disease. |
| 5 | Understanding purposes of home care | Understanding the system of home medical care | physician | For the system to take root, rule making and enforcement are required. |
| 6 | Understanding purposes of home care | Understanding the system of home medical care | nurse | General practitioners sometimes have no transportation to the patient’s home. |
| 7 | An attitude of humility | An attitude of humility | nurse | Respect for the primary physicians’ policies. |
| 8 | An attitude of humility | An attitude of humility | nurse | Understanding what types of treatment are required and focus on urgent care in home-care settings. |
| 9 | An attitude of humility | An attitude of humility | nurse | Understanding the role required for the part-time physicians. |
| 10 | An attitude of humility | An attitude of humility | nurse | Focusing on solving problems consulted for the part-time physicians . |
| 11 | An attitude of humility | An attitude of humility | nurse | The comprehensive ability of the physician in home-care settings. |
| 12 | An attitude of humility | An attitude of humility | physician | The competencies of on-call physician are different from ones of home-care physicians. |
| 13 | An attitude of humility | Pressures on the receiving end of on-call physician | physician | Education in home medical care requires preparation before practice. |
| 14 | An attitude of humility | Pressures on the receiving end of on-call physician | physician | Burden of the main physician accompanied by another physician. |
| 15 | An attitude of humility | Pressures on the receiving end of on-call physician | physician | The inferiority complex of home-care physicians. |
| 16 | Understanding purposes of home care | Understanding purposes of home care | physician | The importance of listening attentively to patients and their families. |
| 17 | Understanding purposes of home care | Understanding purposes of home care | physician | Doctors in the hospital understand the difference of medicine between in-hospital and in-home medical care. |
| 18 | Understanding purposes of home care | Understanding purposes of home care | physician | Limitations of the laboratory and imaging tests that can be performed in home medical care. |
| 19 | Understanding purposes of home care | Understanding purposes of home care | nurse | Be aware that we are going to the patients’ home. |
| 20 | Understanding purposes of home care | Understanding purposes of home care | physician | Put the patient’s life first. |
| 21 | Clinical skills for frequent complaints/Collecting patients' information in advance | Variety of patinets | nurse | Home-care physicians have the time for treating patients with a peritoneal dialysis or intravenous hyperalimentation in home medical care. |
| 22 | Clinical skills for frequent complaints/Collecting patients' information in advance | Variety of patinets | nurse | Home-care physicians often visit patients who live alone. |
| 23 | Clinical skills for frequent complaints/Collecting patients' information in advance | Variety of patinets | nurse | Variety of environments to visit. |
| 24 | Collecting patients' information in advance | Infrequent pre-discharge conferences | physician | Pre-discharge conferences are important but not always possible. |
| 25 | Collecting patients' information in advance | Infrequent pre-discharge conferences | physician | Pre-discharge conferences are infrequent. |
| 26 | Understanding roles of home care health professionals | Expanded scope of practice for nurses | nurse | An increase in the scope of care for home-care nurses. |
| 27 | Understanding roles of home care health professionals | Expanded scope of practice for nurses | physician | An increase in the scope of care for home-care nurses. |
| 28 | Understanding roles of home care health professionals | Expanded scope of practice for nurses | nurse | Possibility of medical procedures performed by skilled nurses with specific training. |
| 29 | Understanding purposes of home care | Distance from the patient in home medical care | nurse | Physicians care for the patients of all ages and have a close relationship with the patients. |
| 30 | Understanding purposes of home care | Distance from the patient in home medical care | nurse | Family care is an important part of home care. |
| 31 | Understanding purposes of home care | Distance from the patient in home medical care | physician | Proximity of home and workplace. |
| 32 | Understanding purposes of home care | Distance from the patient in home medical care | physician | Willingness to conduct urgent home visits. |
| 33 | Understanding purposes of home care | Distance from the patient in home medical care | physician | The scope of care for the primary family physicians. |
| 34 | Understanding purposes of home care | Possible close follow up in home medical care | physician | The importance of regular follow-ups by home-care professionals. |
| 35 | Understanding purposes of home care | Possible close follow up in home medical care | physician | Home-care physicians can modify the practice in regular follow-up. |
| 36 | Understanding purposes of home care | The aptitude of home care physician | physician | Some physicians have concerns regarding their communication skills. |
| 37 | Understanding purposes of home care | The aptitude of home care physician | physician | Some physicians are better suited to work in home medical care. |
| 38 | Understanding purposes of home care | The aptitude of home care physician | physician | The ability to understand people who grew up in different environments. |
| 39 | Understanding purposes of home care | Responding needs as the medical professionals | physician | Different abilities are required in each settings. |
| 40 | Understanding purposes of home care | Responding needs as the medical professionals | physician | During in-home medical care, medical providers may remove stool, change diapers, clean up gastrostomy fluid leaks, and change clothes. |
| 41 | Understanding purposes of home care | Responding needs as the medical professionals | physician | Do what is required for on-call physicians. |
| 42 | Thoughtfulness for patients’ families | Thoughtfulness for patients’ families | nurse | Patients get anxious when they come from different facilities. |
| 43 | Thoughtfulness for patients’ families | Thoughtfulness for patients’ families | nurse | Having on-call physician could help patients feel more secure. |
| 44 | Thoughtfulness for patients’ families | Thoughtfulness for patients’ families | nurse | Patients feel more comfortable when they have an on-call physician from a facility they trust. |
| 45 | Thoughtfulness for patients’ families | Thoughtfulness for patients’ families | nurse | We have to let patients know that we are working together properly. |
| 46 | Thoughtfulness for patients’ families | Thoughtfulness for patients’ families | nurse | We let patients know that we are working properly with the on-call physicians. |
| 47 | Thoughtfulness for patients’ families | Thoughtfulness for patients’ families | physician | The ability to judge an action according to circumstances. |
| 48 | Thoughtfulness for patients’ families | Thoughtfulness for patients’ families | physician | Communication skills are crucial. |
| 49 | Thoughtfulness for patients’ families | Thoughtfulness for patients’ families | physician | The ability to communicate with patients and families. |
| 50 | Thoughtfulness for patients’ families | Thoughtfulness for patients’ families | physician | On-call physicians must have good communication skills. |
| 51 | Thoughtfulness for patients’ families | Thoughtfulness for patients’ families | physician | Understanding patients who tend to be unstable in end-of-life care. |
| 52 | Thoughtfulness for patients’ families | Thoughtfulness for patients’ families | physician | Sharing the patient’s prognosis. |
| 53 | Thoughtfulness for patients’ families | Thoughtfulness for patients’ families | physician | Building relationships with patients. |
| 54 | Thoughtfulness for patients’ families | Thoughtfulness for patients’ families | physician | Assessing the situation through a conversation with the patient. |
| 55 | Thoughtfulness for patients’ families | Thoughtfulness for patients’ families | physician | It is important to reassure patients. |
| 56 | Thoughtfulness for patients’ families | Thoughtfulness for patients’ families | physician | In home care practice, physicians must make decisions based on the five senses and not data. |
| 57 | Thoughtfulness for patients’ families | Thoughtfulness for patients’ families | physician | Touching and examining the patient are crucial. |
| 58 | Thoughtfulness for patients’ families | Thoughtfulness for patients’ families | physician | Because we cannot do a lot of laboratory and imaging tests, a careful physical examination is crucial. |
| 59 | Thoughtfulness for patients’ families | Thoughtfulness for patients’ families | physician | We have to explain so that patients can understand. |
| 60 | Thoughtfulness for patients’ families | Thoughtfulness for patients’ families | physician | The method of explanation and language is important. |
| 61 | Thoughtfulness for patients’ families | Thoughtfulness for patients’ families | nurse | The words of doctors have power. |
| 62 | Thoughtfulness for patients’ families | Thoughtfulness for patients’ families | nurse | Reassuring patients and their families is important. |
| 63 | Thoughtfulness for patients’ families | Thoughtfulness for patients’ families | nurse | The physician’s examination and explanation will convince and reassure patients. |
| 64 | not applicable | Team medical care in home medical care | nurse | Medical supports for home medical care across clinical departments. |
| 65 | not applicable | Team medical care in home medical care | nurse | Diversifying the background of home-care physicians. |
| 66 | not applicable | Team medical care in home medical care | nurse | The team-based practice compensates the expertise of physicians. |
| 67 | not applicable | Team medical care in home medical care | nurse | For patients having specialists as their primary physician, general consultation services may be required. |
| 68 | not applicable | Team medical care in home medical care | nurse | Multiple physicians could treat patients from multiple perspectives. |
| 69 | not applicable | Team medical care in home medical care | nurse | On-call physicians should check with the primary physician when they have questions on the policy. |
| 70 | not applicable | Team medical care in home medical care | nurse | Physicians with little internal medicine experience are also practicing. |
| 71 | not applicable | Team medical care in home medical care | nurse | Team-based care can backup for less-experienced physicians. |
| 72 | not applicable | Team medical care in home medical care | nurse | Team-based care can support physicians from the perspectives of teaching them and sharing their burden for home medical care. |
| 73 | not applicable | Team medical care in home medical care | nurse | Systematize team-based home medical care and match them with suitable patients. |
| 74 | not applicable | Team medical care in home medical care | nurse | A system wherein every patient is assigned a supporting physician. |
| 75 | not applicable | Other needs in home medical care | nurse | If we can consult on-call physicians, we may be able to reduce the number of emergencies. |
| 76 | not applicable | Other needs in home medical care | nurse | Provide care after hours is difficult; therefore, patients have to visit the emergency room. |
| 77 | not applicable | Other needs in home medical care | nurse | If the primary physicians do not provide urgent home medical care, there is a need for on-call physicians. |
| 78 | not applicable | Other needs in home medical care | nurse | Some home-bound patients are supported only by nurses. |
| 79 | not applicable | Other needs in home medical care | nurse | It may be difficult for nurses to provide their home care without physician's orders. |
| 80 | not applicable | Other needs in home medical care | nurse | Responding to minor problems in pediatric patients or patients on respirators. |
| 81 | not applicable | Other needs in home medical care | nurse | It would be helpful if on-call physician could address urgent pediatric patient needs. |
| 82 | not applicable | Other needs in home medical care | nurse | The medical examination of patients with psychiatric disorders from the perspective of internal medicine. |
| 83 | Collecting patients' information in advance | Collecting patients' information in advance | nurse | For some patients, receiving intervention from an on-call physician is difficult. |
| 84 | Collecting patients' information in advance | Collecting patients' information in advance | nurse | There are dangerous situations, and female nurses cannot cope alone. |
| 85 | Collecting patients' information in advance | Collecting patients' information in advance | physician | Building relationships in advance. |
| 86 | Collecting patients' information in advance | Collecting patients' information in advance | nurse | Talking face-to-face is the best way to share information. |
| 87 | Collecting patients' information in advance | Collecting patients' information in advance | physician | Building relationships in advance is important. |
| 88 | Collecting patients' information in advance | Collecting patients' information in advance | physician | Building the relationship before the visit. |
| 89 | Collecting patients' information in advance | Collecting patients' information in advance | physician | Building trust between the primary and on-call physicians. |
| 90 | Collecting patients' information in advance | Collecting patients' information in advance | physician | It is best to accompany main physician on regular visit. |
| 91 | Collecting patients' information in advance | Collecting patients' information in advance | physician | On-call physicians must accompany the primary physician and learn the practice style. |
| 92 | Collecting patients' information in advance | Collecting patients' information in advance | physician | Differences in the practice style of each physician. |
| 93 | Collecting patients' information in advance | Collecting patients' information in advance | physician | Each physician has a different style of medical practice. |
| 94 | Collecting patients' information in advance | Collecting patients' information in advance | physician | On-call physician should learn from each doctor’s practice style. |
| 95 | Collecting patients' information in advance | Collecting patients' information in advance | physician | The difference of the style of medical practice |
| 96 | Collecting patients' information in advance | Collecting patients' information in advance | physician | On-call physician can know the primary physician’s style of medical practice by accompanying them. |
| 97 | Collecting patients' information in advance | Collecting patients' information in advance | nurse | On-call physician should accompany the primary physician when major decisions are made. |
| 98 | Collecting patients' information in advance | Collecting patients' information in advance | nurse | On-call physician can know their style of medical practice by accompanying them. |
| 99 | Collecting patients' information in advance | Collecting patients' information in advance | nurse | On-call physicians have the opportunity to visit the primary physician. |
| 100 | Collecting patients' information in advance | Collecting patients' information in advance | nurse | Importance of meeting the primary physician. |
| 101 | Collecting patients' information in advance | Collecting patients' information in advance | nurse | Create opportunities to interact with patients as a supporting physician. |
| 102 | Collecting patients' information in advance | Collecting patients' information in advance | nurse | Preparation for the supporting physician under the team-based care system. |
| 103 | Collecting patients' information in advance | Collecting patients' information in advance | nurse | Understanding the medical stance of the primary physician. |
| 104 | Collecting patients' information in advance | Collecting patients' information in advance | physician | Sharing medical perspective with patients. |
| 105 | Collecting patients' information in advance | Collecting patients' information in advance | physician | Importance of advance prediction. |
| 106 | Collecting patients' information in advance | Collecting patients' information in advance | physician | Description for sharing information on the diagnosis, patient’s condition, Activities of Daily Living, caregiving capacity, and policy in case of emergency. |
| 107 | Collecting patients' information in advance | Collecting patients' information in advance | physician | The policy in case of emergency is crucial. |
| 108 | Collecting patients' information in advance | Collecting patients' information in advance | nurse | If the primary physician is absent, on-call physician must discuss the anticipated situation in advance. |
| 109 | Collecting patients' information in advance | Collecting patients' information in advance | nurse | The family situation and personality of the patient are also important. |
| 110 | Collecting patients' information in advance | Collecting patients' information in advance | nurse | Prior information sharing among physicians. |
| 111 | Collecting patients' information in advance | Collecting patients' information in advance | nurse | Information to be shared. |
| 112 | Collecting patients' information in advance | Collecting patients' information in advance | nurse | Name of disease, medication, family structure, primary caregiver, treatment plan, prognosis, patient’s decision-making process and wishes for end-of-life care. |
| 113 | Collecting patients' information in advance | Collecting patients' information in advance | nurse | Care services must also be shared. |
| 114 | Collecting patients' information in advance | Collecting patients' information in advance | physician | It is difficult to update accurate information on medications. |
| 115 | Collecting patients' information in advance | Collecting patients' information in advance | nurse | Including the medication name in the home nursing instructions was important. |
| 116 | Collecting patients' information in advance | Collecting patients' information in advance | physician | Home-care nurses keep track of medication information. |
| 117 | Collecting patients' information in advance | Collecting patients' information in advance | nurse | There must be a clear intention in requesting an emergency visit. |
| 118 | Collecting patients' information in advance | Collecting patients' information in advance | nurse | Accurate communication of information. |
| 119 | Collecting patients' information in advance | Collecting patients' information in advance | nurse | The clarification of a backup system and information sharing with home nursing stations. |
| 120 | Collecting patients' information in advance | Collecting patients' information in advance | physician | Information sharing on electronic systems. |
| 121 | Collecting patients' information in advance | Collecting patients' information in advance | physician | I think it would be a good idea to share information via the web. |
| 122 | Collecting patients' information in advance | Collecting patients' information in advance | physician | Sharing patient information via the web. |
| 123 | Understanding roles of home care health professionals | Understanding roles of home care health professionals | physician | Gathering information from home-care nurses. |
| 124 | Understanding roles of home care health professionals | Understanding roles of home care health professionals | physician | Gathering information about the primary physician’s style of practice from the home-care nurse. |
| 125 | Understanding roles of home care health professionals | Understanding roles of home care health professionals | physician | Home-care nurses get the first call. |
| 126 | Understanding roles of home care health professionals | Understanding roles of home care health professionals | physician | The nurse listens to the family’s thoughts. |
| 127 | Understanding roles of home care health professionals | Understanding roles of home care health professionals | physician | The nurse could report what she has done after the first call. |
| 128 | Understanding roles of home care health professionals | Understanding roles of home care health professionals | physician | Understanding the medical attitude of home-care nurses. |
| 129 | Understanding roles of home care health professionals | Understanding roles of home care health professionals | nurse | Communication with home-care nurses is crucial. |
| 130 | Understanding roles of home care health professionals | Understanding roles of home care health professionals | physician | Information sharing with home-care nurse stations. |
| 131 | Understanding roles of home care health professionals | Understanding roles of home care health professionals | physician | More information is shared with home-care nurses than with doctors. |
| 132 | Understanding roles of home care health professionals | Understanding roles of home care health professionals | physician | Differences in response among home-care nurse stations. |
| 133 | Understanding roles of home care health professionals | Understanding roles of home care health professionals | nurse | Sometimes home-care workers are the most trusted people for patients. |
| 134 | An attitude of humility | Relationship with the main physicians | physician | Cooperation from the primary physician. |
| 135 | An attitude of humility | Relationship with the main physicians | physician | The relationship with the primary physician is further important. |
| 136 | Clinical skills for frequent complaints | Clinical skills for frequent complaint | nurse | Fever, abnormal blood pressure, dehydration, and heat strokes. |
| 137 | Clinical skills for frequent complaints | Clinical skills for frequent complaint | physician | Fever (urinary tract infection and pneumonia) is a common complaint. |
| 138 | Clinical skills for frequent complaints | Clinical skills for frequent complaint | physician | Fever is a common patient complaint in an emergency visit. |
| 139 | Clinical skills for frequent complaints | Clinical skills for frequent complaint | physician | The frequency of fever cause. |
| 140 | Clinical skills for frequent complaints | Clinical skills for frequent complaint | physician | What is required of the on-call physician is different from that of the primary physician. |
| 141 | Clinical skills for frequent complaints | Clinical skills for frequent complaint | nurse | Decisions and instructions for dehydration, pressure ulcers, infection, dyspnea, falls, and fractures. |
| 142 | Clinical skills for frequent complaints | Clinical skills for frequent complaint | nurse | Focusing on the needs of frequent complaints, such as infection, dehydration, and pressure ulcers. |
| 143 | Clinical skills for frequent complaints | Clinical skills for frequent complaint | physician | The frequency of visits depends on the patient’s condition. |
| 144 | Clinical skills for frequent complaints | Clinical skills for frequent complaint | nurse | Patients with mental illnesses make numerous phone calls. |
| 145 | Clinical skills for frequent complaints | Clinical skills for frequent complaint | nurse | Naso-gastric tube and urinary catheter insertion are some of the techniques required. |
| 146 | Clinical skills for frequent complaints | Clinical skills for frequent complaint | nurse | Home-care physicians receive numerous calls on problems with catheter systems. |
| 147 | Clinical skills for frequent complaints | Clinical skills for frequent complaint | nurse | The treatment of pain and fever at home is required. |
| 148 | Clinical skills for frequent complaints | Clinical skills for frequent complaint | physician | On-call physicians are not expected to do a lot of work but to only get through the situation. |
| 149 | Clinical skills for frequent complaints | Clinical skills for frequent complaint | physician | On-call physicians have to get through the situation. |
| 150 | Clinical skills for frequent complaints | Clinical skills for frequent complaint | nurse | The family wants the doctor to take care of the patient at the end. |
| 151 | Clinical skills for frequent complaints | Clinical skills for frequent complaint | nurse | The question arises as to who will write the death certificate. |
| 152 | Clinical skills for frequent complaints | Clinical skills for frequent complaint | nurse | The secondary physician system is likely to be used in the end-of-life-care situations. |
| 153 | Clinical skills for frequent complaints | Clinical skills for frequent complaint | nurse | The confirmation of death if the primary physician is unable to come. |
| 154 | Clinical skills for frequent complaints | Clinical skills for frequent complaint | physician | Many calls for the confirmation of death. |
| 155 | Clinical skills for frequent complaints | Clinical skills for frequent complaint | physician | Death certification could be expected and unexpected. |
| 156 | Clinical skills for frequent complaints | Clinical skills for frequent complaint | physician | No significant difference is observation between the end-of-life care at hospitals and home. |
| 157 | Clinical skills for frequent complaints | Clinical skills for frequent complaint | physician | What I want to ask for the supporting physician is the death certification. |
| 158 | Clinical skills for frequent complaints | Clinical skills for frequent complaint | nurse | Home-care physicians receive numerous calls from terminally ill patients. |
| 159 | Clinical skills for frequent complaints | Clinical skills for frequent complaint | nurse | Terminally ill patients and their caregivers call a lot. |
| 160 | Understanding purposes of home care | The request of main physician to on-cal physician | physician | The time I ask for on-call physician is when I am on vacation. |
| 161 | Understanding purposes of home care | The request of main physician to on-cal physician | physician | I only have to respond to an emergency call at night approximately once a month. |
| 162 | Understanding purposes of home care | The request of main physician to on-cal physician | physician | The frequency of emergency calls at night. |
| 163 | Understanding roles of home care health professionals | Triage of home visits by nurses | physician | Home-care nurses determine the need for an emergent visit. |
| 164 | Understanding roles of home care health professionals | Triage of home visits by nurses | nurse | Home-care nurses received calls approximately three times a week. |
| 165 | Understanding roles of home care health professionals | Triage of home visits by nurses | nurse | Only approximately 10% of emergent calls to home-care nurses are connected to physicians. |
